# Cerebral Blood Flow Autoregulation in Offspring From Experimentally Preeclamptic Rats and the Effect of Age

**DOI:** 10.3389/fphys.2022.924908

**Published:** 2022-06-06

**Authors:** Emmett E. Whitaker, Abbie C. Johnson, Sarah M. Tremble, Conor McGinn, Nicole DeLance, Marilyn J. Cipolla

**Affiliations:** ^1^ Department of Anesthesiology, University of Vermont Larner College of Medicine, Burlington, VT, United States; ^2^ Department of Neurological Sciences, University of Vermont Larner College of Medicine, Burlington, VT, United States; ^3^ Department of Pediatrics, University of Vermont Larner College of Medicine, Burlington, VT, United States; ^4^ Department of Pathology and Laboratory Medicine, University of Vermont Larner College of Medicine, Burlington, VT, United States; ^5^ Department of Obstetrics, Gynecology, and Reproductive Sciences, University of Vermont Larner College of Medicine, Burlington, VT, United States; ^6^ Department of Pharmacology, University of Vermont Larner College of Medicine, Burlington, VT, United States; ^7^ University of Vermont Department of Electrical and Biomedical Engineering, Burlington, VT, United States

**Keywords:** cerebral blood flow (CBF), offspring, pregnancy, autoregulation, aging, preeclampsia

## Abstract

Preeclampsia is a hypertensive disorder of pregnancy that causes significant, long term cardiovascular effects for both the mother and offspring. A previous study demonstrated that middle cerebral arteries in offspring from an experimental rat model of preeclampsia were smaller, stiffer, and did not enlarge over the course of maturation, suggesting potential hemodynamic alterations in these offspring. Here we investigated the effect of experimental preeclampsia on cerebral blood flow autoregulation in juvenile and adult offspring that were born from normal pregnant or experimentally preeclamptic rats. Relative cerebral blood flow was measured using laser Doppler flowmetry, and cerebral blood flow autoregulation curves were constructed by raising blood pressure and controlled hemorrhage to lower blood pressure. Immunohistochemistry was used to assess middle cerebral artery size. Heart rate and blood pressure were measured in awake adult offspring using implanted radiotelemetry. Serum epinephrine was measured using enzyme-linked immunosorbent assay. Offspring from both groups showed maturation of cerebral blood flow autoregulation as offspring aged from juvenile to adulthood as demonstrated by the wider autoregulatory plateau. Experimental preeclampsia did not affect cerebral blood flow autoregulation in juvenile offspring, and it had no effect on cerebral blood flow autoregulation in adult offspring over the lower range of blood pressures. However, experimental preeclampsia caused a right shift in the upper range of blood pressures in adult offspring (compared to normal pregnant). Structurally, middle cerebral arteries from normal pregnant offspring demonstrated growth with aging, while middle cerebral arteries from experimentally preeclamptic offspring did not, and by adulthood normal pregnant offspring had significantly larger middle cerebral arteries. Middle cerebral artery lumen diameters did not significantly change as offspring aged. Serum epinephrine was elevated in juvenile experimentally preeclamptic offspring, and a greater degree of hemorrhage was required to induce hypotension, suggesting increased sympathetic activity. Finally, despite no evidence of increased sympathetic activity, adult experimentally preeclamptic offspring were found to have persistently higher heart rate. These results demonstrate a significant effect of experimental preeclampsia on the upper range of autoregulation and cerebrovascular structure in juvenile and adult offspring that could have an important influence on brain perfusion under conditions of hypo and/or hypertension.

## 1 Introduction

Preeclampsia (PE) is a devastating hypertensive disorder that complicates 5–10% of pregnancies worldwide (Wallis et al., 2008; Duley, 2009; Hutcheon et al., 2011). PE creates an unfavorable intrauterine environment for the fetus that is characterized by chronic inflammation, oxidative stress, hypoxia, and placental insufficiency that leads to long-term cardiovascular consequences for both mother and baby (Friedman et al., 1995; Stark et al., 2009; Geelhoed et al., 2010; Backes et al., 2011; Fugelseth et al., 2011; Dang et al., 2016; Giachini et al., 2017; Gumusoglu et al., 2020). Offspring of preeclamptic pregnancies have been shown to have an increased risk of stroke over the entire lifespan as well as early-onset hypertension, suggesting the presence of a cardiovascular disease risk profile that presents early and persists into adulthood (Kajantie et al., 2009; Oglaend et al., 2009; Geelhoed et al., 2010). However, relatively little is known about the effect of PE on cerebrovascular function in young offspring or the influence of maturation, both of which may provide insight into disease development and progression. In addition, underlying mechanisms by which PE adversely affects the cerebral vasculature of offspring are not well-studied.

Cerebral blood flow autoregulation (CBFAR) is the innate ability of the brain to maintain a relatively constant blood flow over a range of cerebral perfusion pressures. This characteristic of cerebrovascular physiology is critical in maintaining adequate brain perfusion during periods when blood pressure changes from baseline (e.g., during acute hypotension or hypertension). The traditional model of CBFAR described by Lassen in 1959 was later interpreted to indicate that CBFAR can maintain constant blood flow over a wide range of mean arterial pressures (e.g., 50–150 mmHg in adults) (Lassen, 1959). This concept suggests that CBFAR has limits, that is, above and below certain pressures, CBF changes linearly with pressure. Though it is now known that CBFAR is much more complex and nuanced, this concept is still used clinically (Claassen et al., 2021).

Disease states such as chronic hypertension, stroke, and traumatic brain injury are known to lead to impairment of CBFAR (Rangel-Castilla et al., 2008; Aries et al., 2010; Cipolla et al., 2011; van Veen et al., 2013; Shekhar et al., 2017; Jones and Warrington, 2019). Pregnancy has also been shown to cause significant changes in maternal cerebral hemodynamics, including decreased cerebral blood flow (CBF) velocity, increased cerebral blood volume, and decreased cerebrovascular resistance (Nevo et al., 2010; van Veen et al., 2016). Further, PE is known to impair CBFAR in pregnant women and animal models (Cipolla et al., 2011; van Veen et al., 2013). Outside of pregnancy, this impairment has detrimental consequences for the brain and can progress to secondary injury, including blood brain barrier damage, cerebral edema, and neurodegeneration (Cipolla, 2007; Cipolla, 2013; Hammer and Cipolla, 2015; Siepmann et al., 2017; Clayton et al., 2018). It remains unclear, however, how *in utero* exposure to PE affects CBFAR in offspring, particularly in very young offspring. Understanding how fetal exposure to PE affects CBFAR is important because it may increase the susceptibility of the brain to either hypoperfusion or hyperperfusion injury during times of hypo- or hypertension (e.g., during surgery, sepsis, or critical illness), respectively.

Maintaining adequate cerebral perfusion is paramount to avoiding brain injury, particularly during times of hypotension that may be persistent or occurs frequently. PE-exposed offspring are likely to present for clinical care at one or more points over the course of their lifespan, and episodes of hyper- and hypotension can be expected in these patients (Geelhoed et al., 2010; Alsnes et al., 2017). For example, offspring from PE pregnancies are more likely to have congenital heart disease and stroke (Curry et al., 2007; Kajantie et al., 2009), both of which are associated with systemic hemodynamic instability. For this reason, understanding how exposure to PE affects CBFAR in offspring is critical to guide intervention when perturbations in blood pressure occur.

In the current study, we compared CBFAR and factors that affect it in both juvenile and adult offspring of experimentally preeclamptic (ePE) rats. We hypothesized that CBFAR would be impaired, *via* an increase in the lower limit of autoregulation and/or a decrease in the upper limit of autoregulation, in both juvenile and adult offspring from ePE rats. In addition, we investigated sympathetic nervous system activation (serum epinephrine) and cerebral artery remodeling (MCA medial and lumen area) as potential underlying mechanisms of ePE-induced changes in CBFAR.

## 2 Methods

### 2.1 Statement of Ethics

All procedures were approved by the Institutional Animal Care and Use Committee at the University of Vermont and complied with the National Institutes of Health guidelines for care and use of laboratory animals. Studies were conducted in accordance with Animal Research: Reporting of *In Vivo* Experiments (ARRIVE-2) guidelines (Kilkenny et al., 2010).

### 2.2 Animals and Induction of ePE

Pregnant Sprague-Dawley rats were housed singly with environmental enrichment in the University of Vermont Animal Care Facility, an Association for Assessment and Accreditation of Laboratory Animal Care International (AAALAC) accredited facility. Rats were maintained on a 12-h light/dark cycle and allowed access to food and water ad libitum. ePE was induced in dams by administering a high-cholesterol diet beginning on day 7 of gestation as previously described (Schreurs et al., 2013; Schreurs and Cipolla, 2014; Johnson and Cipolla, 2017; Johnson and Cipolla, 2018). This model has been previously shown to induce several hallmark symptoms of PE, including maternal dyslipidemia, endothelial dysfunction, systemic inflammation, decreased placental weight, and increased blood pressure (Schreurs and Cipolla, 2013; Schreurs et al., 2013). This model is also associated with fetal growth restriction suggesting it induces impaired uteroplacental blood flow and/or placental disease associated with PE (Schreurs et al., 2013).

### 2.3 Offspring

Offspring of normal pregnant (NP) and ePE rats were born in our animal facility and were used for experimentation at postnatal day 27–30 (p27–30, juvenile) or 18–22 weeks old (adult). Offspring of either sex were randomly selected using a coin flip. ePE dams were maintained on a high-cholesterol diet, and male and female offspring were weaned at p21. Offspring were housed in groups and fed regular rat chow until experimental use. All offspring were weighed immediately prior to experimentation.

### 2.4 CBFAR Experiments

Juvenile and adult offspring were anesthetized with 3% isoflurane in oxygen and then endotracheally intubated and mechanically ventilated with a mixture of oxygen and air to maintain blood gases and pH in normal physiologic ranges. Catheters were placed in the femoral arteries and veins to administer pharmacological agents, continuously measure arterial blood pressure (BP) and to induce hypotension *via* controlled hemorrhage. Following placement of catheters, anesthesia was transitioned to i.v. chloral hydrate (50 mg/ml, 200–300 mg‧kg^−1^ total dose) with simultaneous off-titration of isoflurane. Rats remained on chloral hydrate for the remainder of the experiment. Chloral hydrate was selected because unlike isoflurane, which is a potent cerebral vasodilator that could confound CBF measurements, chloral hydrate has minimal effects on cerebral hemodynamics (Nakao et al., 2001; Wang et al., 2010).

Laser Doppler flowmetry (Perimed Inc., Ardmore, PA, United States) was used to continuously measure relative changes in CBF in the territory of the middle cerebral artery (MCA) as previously described (Cipolla et al., 2017). Briefly, a midline scalp incision was made, and the scalp gently retracted. A laser Doppler probe was placed in the MCA territory (+3 mm lateral and −1 mm posterior to Bregma for juvenile; +4 mm lateral and −2 mm posterior to Bregma for adult) as described in rat brain atlases (Buttner-Ennever, 1997; Khazipov et al., 2015) and secured in place with superglue. After placement of the laser Doppler probe, appropriate depth of anesthesia was assured, and blood pressure was allowed to stabilize. Baseline flow was recorded for 5 minutes (baseline CBF measurement).

To determine the upper limit of autoregulation, norepinephrine (NE, 2.5 mg/ml) was continuously infused *via* femoral venous catheter to increase BP in 10 mmHg increments. The initial NE rate of infusion was 1 μL/min. NE infusion was increased 1 μL/min at a time until BP increased 10 mmHg and the next pressure step was reached. BP was allowed to stabilize at each pressure step for approximately 5 min before proceeding to the next pressure step. The pressure at which increasing doses of NE no longer increased BP was considered maximum BP. Once this was reached, NE infusion was discontinued, and BP was allowed to return to baseline for at least 10 min.

To determine the lower limit of CBF autoregulation, blood was slowly removed *via* a femoral arterial catheter to decrease BP in 10 mmHg increments. BP was allowed to stabilize at each pressure step for approximately 5 minutes before proceeding to the next pressure step. Once the minimum BP of 20 mmHg was reached, hemorrhage was stopped. The volume of blood removed to achieve each pressure step was recorded and serum was recorded for later analysis.

#### 2.4.1 Determination of Middle Cerebral Artery Size

Following CBFAR experiments, animals were decapitated under anesthesia. Brains were removed and immediately placed in ice-cold, freshly bubbled physiologic saline solution (PSS). MCAs were dissected and fixed in 10% buffered formalin at 4°C overnight then paraffin embedded for immunohistochemistry. For each animal, two adjacent 3 μm thick sections were cut on a Leica RM2145 paraffin microtome (Leica Microsystems, Buffalo Grove, IL) and retrieved onto slides. Slides were air dried overnight and baked at 60°C for 1 hour prior to staining. Sections were deparaffinized in three changes of xylene and rehydrated through graded ethanol. For immunofluorescence, antigen retrieval was performed using DAKO Target Retrieval Solution (pH 6.0) at 96°C, whereafter slides were blocked in 5% BSA/10% normal goat serum. Rabbit polyclonal matrix metalloproteinase-9 (MMP-9), represented in red, (Invitrogen # MA5-32705) and mouse monoclonal elastin, represented in green, (Abcam # ab77804) were applied to vessels at 1:200 and 1:100, respectively, overnight at 4°C, followed by rinses with buffer. Elastin was used to identify the inner boundary of the medial layer, and MMP-9 was used to visualize the medial layer for quantification. Presence of bound primary antibody was detected using Invitrogen goat anti-rabbit IgG Alexa Fluor 555 secondary antibody, and goat anti-mouse IgG Alexa Fluor 647 secondary antibody. Finally, the nuclei were counterstained with DAPI and coverslipped in aqueous mounting media. Sections were imaged on a Nikon A1R-ER Confocal Microscope using a Plan Fluor 40x Oil DIC H N2, NA 1.3, WD = 240 µm objective lens. Images were captured at ×40 magnification from each MCA using NIS-Elements Ar (version 4.30.02; Nikon United States, Tokyo, Japan). Medial area and lumen area were quantified from the 40X images using NIS Elements software. Images were analyzed by an investigator blinded to group.

#### 2.4.2 Data Calculations

For CBFAR, percent change in blood flow was calculated for each BP step according to the following equation:

$$CBF\;Percent\;Change=\left[\frac{CBF\;at\;Pressure-CBF\;at\;Baseline}{CBF\;at\;Baseline}\right]x100\%$$ (1)

Upper and lower limits of autoregulation were defined as the blood pressure at which relative CBF decreased (lower limit) or increased (upper limit) by 20%. This threshold was chosen because sustained decreases in CBF of 20% or greater are known to cause ischemia within minutes (Astrup et al., 1981; Siesjö, 1992; Lipton, 1999).

### 2.5 Radiotelemeter Implantation

To continuously measure heart rate (HR) and BP, physiologic telemetry transmitters were implanted in a separate set of adult offspring from the same dams as previously described (Daubert et al., 2014; Erdos et al., 2015). Due to the size of the transmitters, they could only be safely implanted in adult offspring. Briefly, telemeters (model PA-C40; Data Sciences International, St. Paul, MN) were implanted into the descending aorta under isoflurane anesthesia. A midline incision was made, and the abdominal aorta was isolated. The aorta was briefly occluded, and the telemeter catheter was inserted into the aorta using a 21-gauge needle. A nitrocellulose patch and surgical glue (Vetbond Tissue Adhesive, 3M, St. Paul, MN, USA) were used to secure the catheter in place. The telemeter was sutured to the abdominal muscles. Buprenorphine (0.05 mg‧kg^−1^ subcutaneously) was administered at 0, 3, and 24 h postoperatively for analgesia.

Offspring were allowed to recover for 2 weeks before data collection. HR and BP data were collected using Dataquest A.R.T. analysis software (Data Sciences International). Over a period of 5 days, data were collected for 15 s every 10 min and averaged per hour. Data collected between 8 a.m. and 4 p.m. were averaged to calculate diurnal values, whereas data collected between 8 p.m. and 4 a.m. were averaged to calculate nocturnal values for each animal.

Variability in HR was assessed by calculating the standard deviation of the 10-min averages for each group (NP vs. ePE) over the course of the last 24 h of telemetry. Variability was compared every 10 min over the entire 24 h period, and the average variability for the entire 24 h period was calculated and compared.

### 2.6 Serum Analysis

Serum samples were obtained *via* femoral artery catheter during CBFAR experiments and collected in serum separator tubes. After 30 min, tubes were centrifuged for 10 min at 2,500 revolutions per minute at room temperature, and serum immediately aliquoted in 200 µL microcentrifuge tubes and stored at −80°C until measurement. Serum levels of epinephrine were measured in duplicate using a commercially available enzyme-linked immunosorbent assay (ELISA) kit (LSBio, Seattle, WA) according to manufacturer instructions. Serum levels of norepinephrine were measured in duplicate using a commercially available ELISA kit (myBioSource, San Diego, CA) according to manufacturer instructions. Undiluted samples were run in duplicate.

### 2.7 Data Analysis and Statistical Considerations

The number of animals used in each experiment was justified by a statistical power calculation based on previous studies using similar methodology (Cipolla et al., 2012). All data were analyzed by an investigator blinded to study group. Data are presented as mean ± SEM. Differences between two groups in CBFAR, HR, HR variability, serum levels of epinephrine/norepinpehrine, and blood volume removal were determined using Student’s t-tests or Mann-Whitney tests (for non-normally distributed data). To determine the effect of two independent variables (age and ePE) on MCA structure (four groups), a two-way analysis of variance (ANOVA) with Tukey test for multiple comparisons was used. Statistics were performed using GraphPad Prism (Version 9.0.0, GraphPad Software, San Diego, CA, United States) and differences were considered significant when *p* < 0.05.

## 3 Results

### 3.1 Effect of ePE on CBFAR

A mix of male and female offspring were used for each experiment as described in the methods section. Male-female distribution for each experimental group is provided in each figure legend. Offspring from NP and ePE rats were found to have similar body weight at juvenile ages (98.8 ± 16.5 g vs. 114.6 ± 14.4 g; *p* > 0.05 by Mann-Whitney test) and in adulthood (435.9 ± 105.6 g vs. 542.9 ± 154.3 g; *p* > 0.05 by Mann-Whitney test).

**FIGURE 1:**
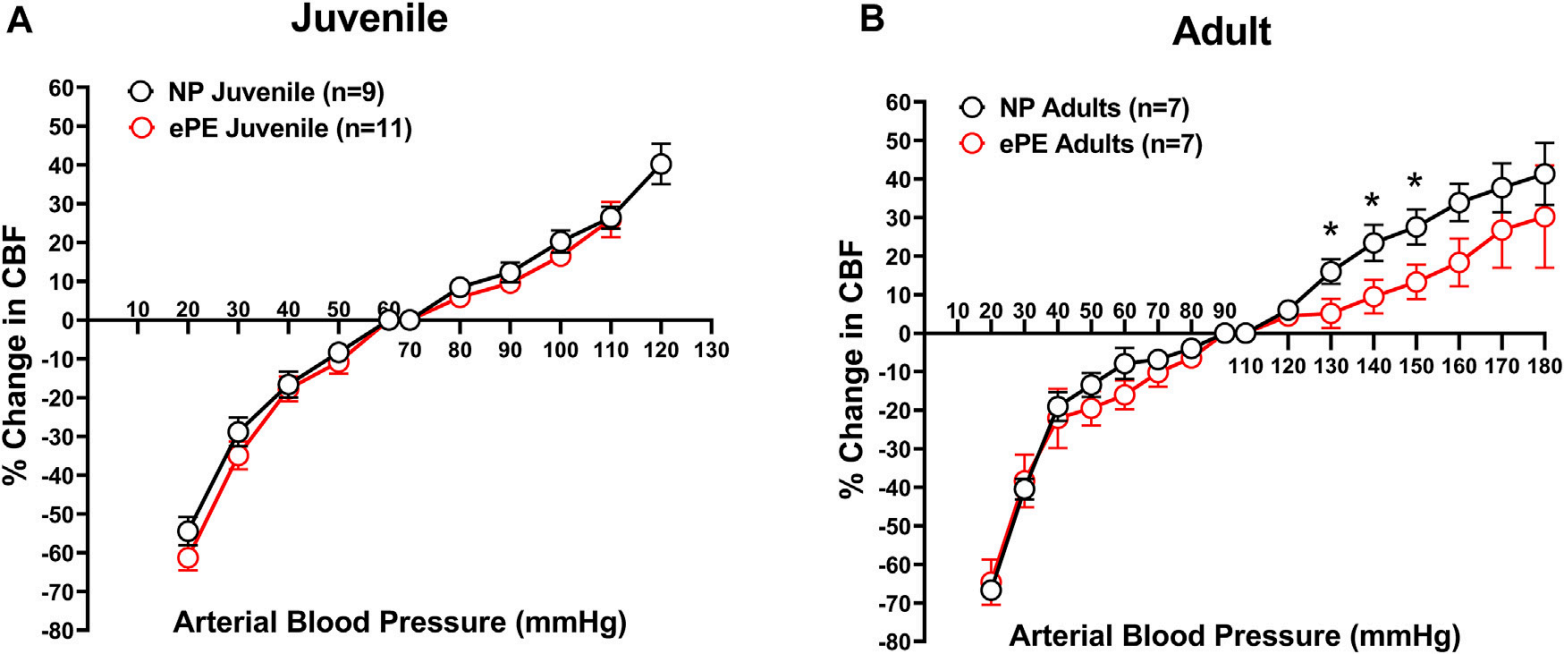
CBFAR curves for p30 and adult offspring of NP vs. ePE dams. **(A)**, there was no difference in CBFAR in p30 offspring between groups. **(B)**, CBFAR in adult offspring from ePE dams was extended to higher pressures. NP juvenile: *n* = 9 (5 male, 4 female); ePE juvenile: *n* = 11 (6 male, 5 female); NP adult: *n* = 7 (3 male, 4 female); ePE adult: *n* = 7 (4 male, 3 female). *p < 0.05, Student’s t-test.

To determine the effect of ePE on CBFAR in offspring, and whether this changed with age, CBFAR curves were determined for both juvenile and adult offspring from NP and ePE rats (Figures 1A,B). CBFAR curves in juvenile offspring were similar between NP and ePE pregnancies. In adult offspring, CBFAR curves were similar within the lower range of pressures (20–100 mmHg). However, there was a right shift in the upper range of the CBFAR curve in adult ePE offspring compared to NP offspring. This rightward shift was characterized by a significantly lower % change in CBF in ePE offspring at BPs of 130, 140, and 150 mmHg (*p* < 0.05).

**FIGURE 2:**
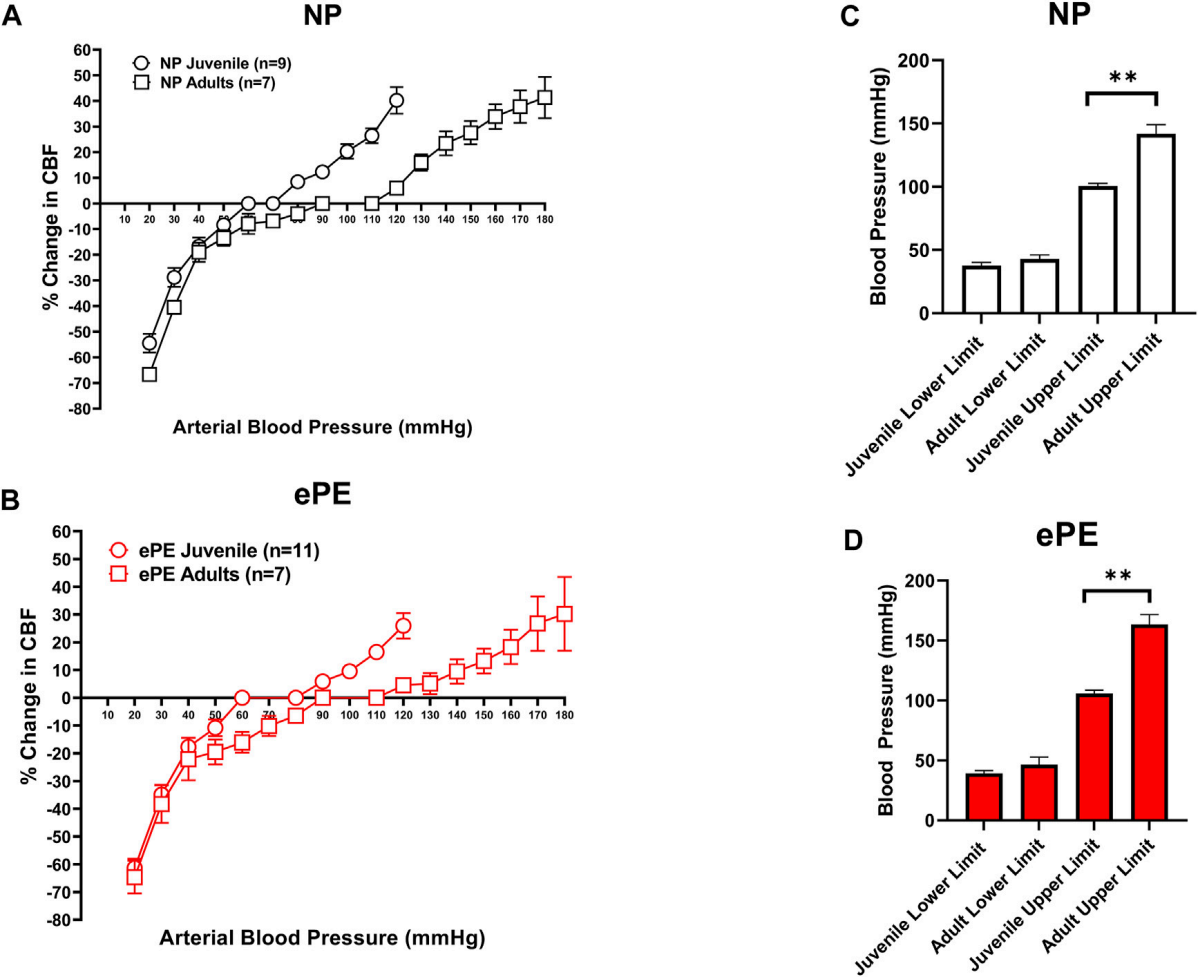
Comparison of CBFAR curves between p30 and adult offspring. CBFAR curves were not different at the lower pressures but were extended towards higher pressures in adults vs. p30 for offspring from both NP **(A)** and ePE **(B)**. The lower limits of CBFAR were not different between adult and p30 in either group. However, the upper limit was extended in adults from both groups **(C,D)**. NP juvenile: *n* = 9 (5 male, 4 female); ePE juvenile: *n* = 11 (6 male, 5 female); NP adult: *n* = 7 (3 male, 4 female); ePE adult: *n* = 7 (4 male, 3 female). *p < 0.05, Mann-Whitney test.

Figures 2A,B compare CBFAR curves from juvenile vs. adult offspring within group (NP and ePE). In both the NP and ePE groups, autoregulation curves were similar over the lower range of blood pressures. However, CBFAR curves were shifted to the right in the upper range in adult offspring from both NP and ePE rats. Figures 2C,D show upper and lower limits of autoregulation, defined as the pressure at which relative CBF rose or fell 20%. Neither the upper nor lower limits of autoregulation were found to be different when ePE and NP offspring were compared. Additionally, lower limits of autoregulation were similar when juvenile and adult offspring were compared regardless of group. However, when compared to juvenile offspring, the upper limit of autoregulation was found to be significantly higher in adult offspring for both the NP (101 vs. 142 mmHg, *p* < 0.01) and ePE (106 vs. 163 mmHg, *p* < 0.01) groups compared to juvenile offspring. These data suggest that aging shifts the upper, but not lower, limit of autoregulation to a higher blood pressure in both NP and ePE offspring. Limits of CBFAR between NP and ePE for both juvenile and adult offspring were also compared. Though there was no statistically significant difference in upper or lower limits for either group, the difference in the upper limit of autoregulation for NP vs. ePE adult offspring trended toward being higher in adult offspring from ePE pregnancies when compared to adult offspring from NP pregnancies (*p* = 0.10, Student’s t-test).

### 3.2 Hemorrhage Required to Induce Hypotension

**FIGURE 3:**
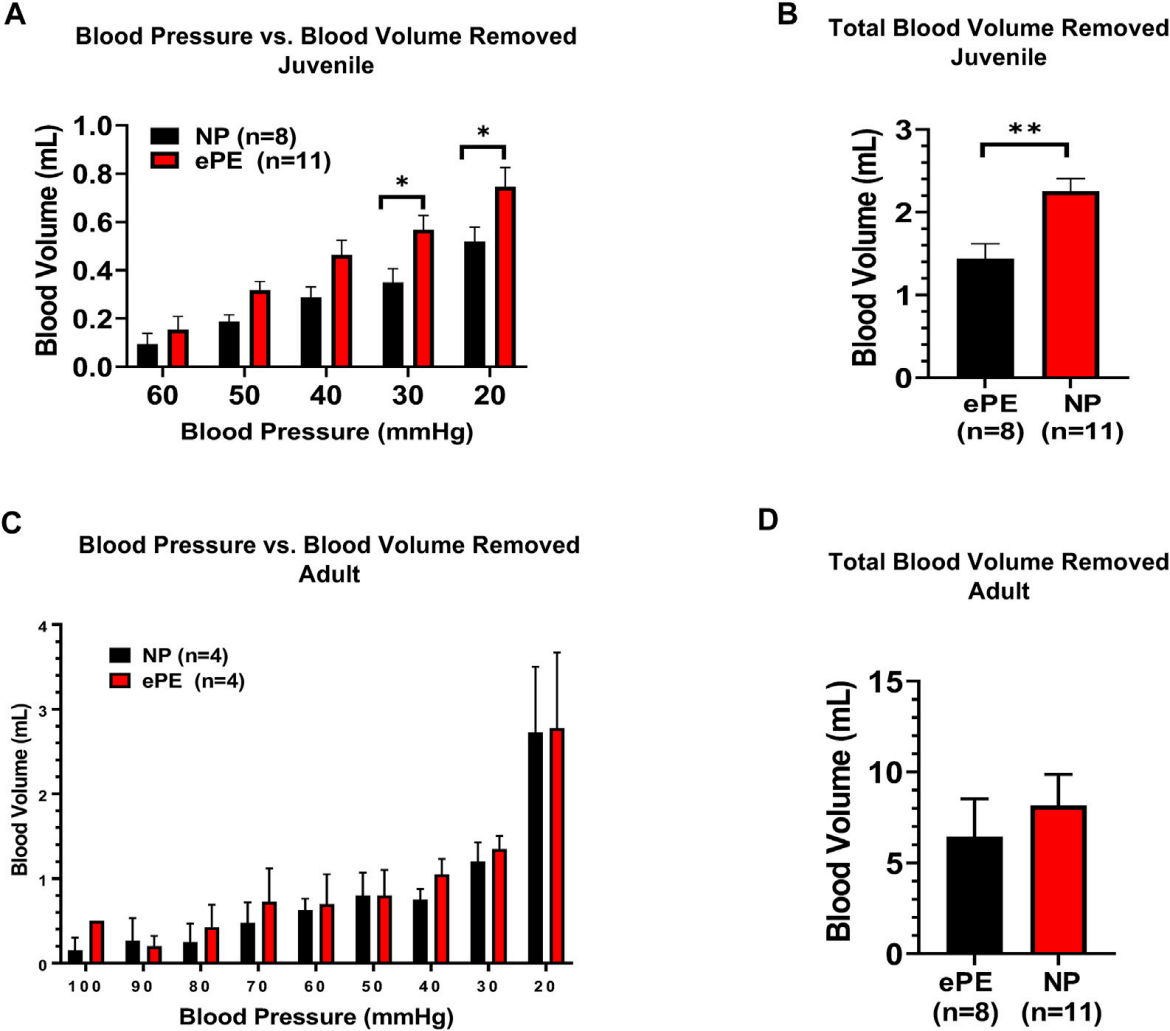
Blood volume removed during hemorrhagic hypotension to reach target blood pressures. **(A)**, significantly greater blood volume removal was needed to reach pressures of 30 and 20 mmHg in juvenile offspring from ePE rats compared to NP. **(B)**, total blood volume removed to reach the lower limit of 20 mmHg from baseline was greater in juvenile offspring from ePE dams. **(C,D)**, there was no difference in the volume of blood removal needed to reach individual pressures or total blood volume from baseline in adult offspring from either group. **p < 0.01; *p < 0.05, Student’s t-test.

Figure 3 summarizes the volume of hemorrhage required to reduce blood pressure in 10 mmHg increments. Figure 3A shows that in juvenile offspring, it was necessary to remove more blood to induce hypotension in offspring from ePE dams at 30 mmHg (0.35 vs. 0.57 ml, *p* < 0.05) and 20 mmHg (0.52 vs. 0.75 ml, *p* < 0.05). In addition, the total amount of hemorrhage required to reach a sustained blood pressure of 20 mmHg was significantly higher in ePE juvenile offspring (Figure 3B, 1.44 vs. 2.25 ml, *p* < 0.01). In contrast, there was no significant difference in the amount of hemorrhage required to reach any blood pressure (Figure 3C) nor was there a difference in the total amount of hemorrhage required (Figure 3D) when adult offspring from NP vs. ePE rats were compared.

### 3.3 Serum Epinephrine and Norepinephrine

**FIGURE 4:**
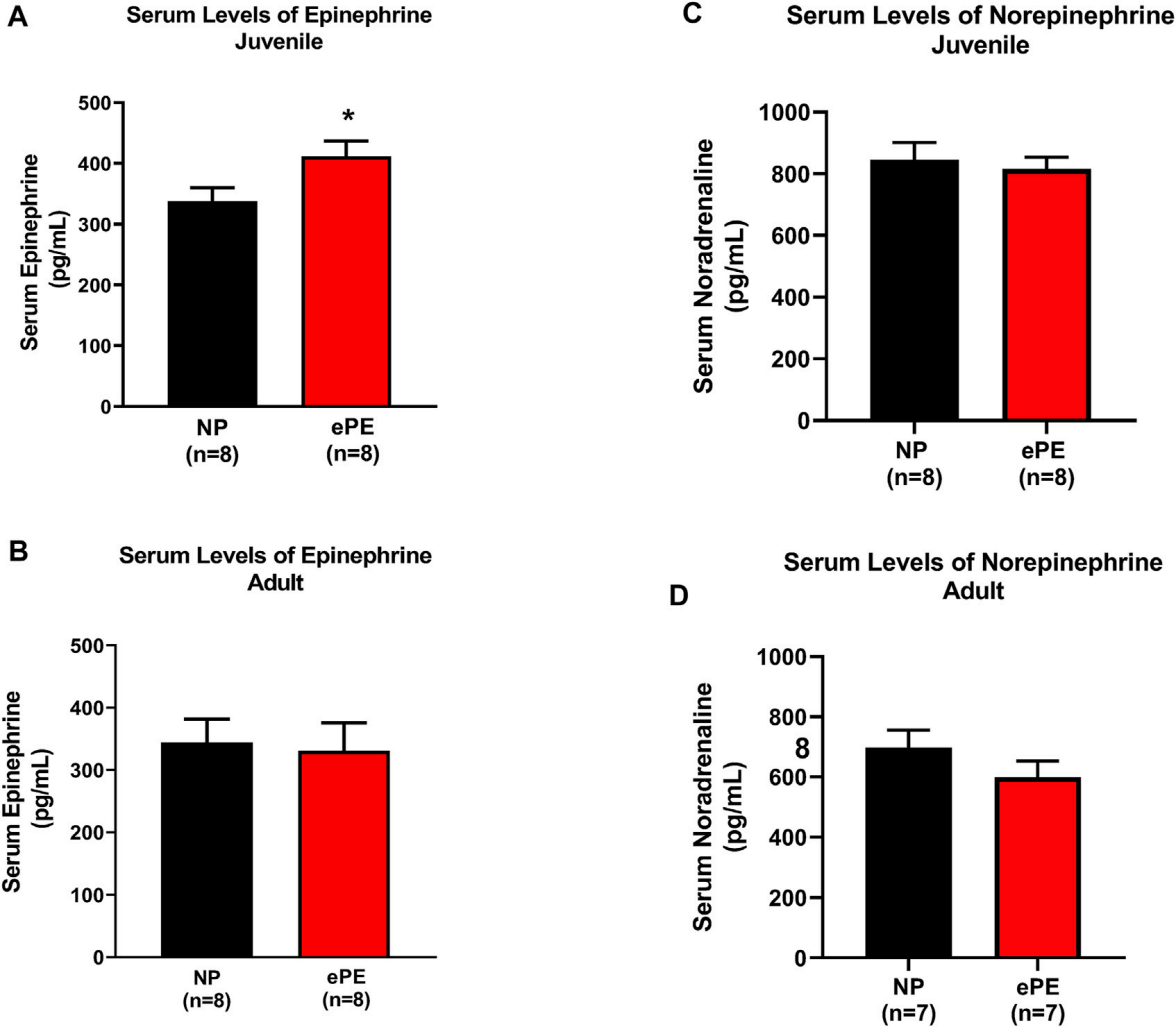
Serum epinephrine and norepinephrine concentrations in NP vs. ePE offspring. **(A,B)**, juvenile but not adult ePE offspring showed higher serum epinephrine concentrations. **(C,D)** There was no difference in serum norepinephrine from either juvenile or adult offspring, NP vs. ePE. NP juvenile: *n* = 8 (4M, 4 female); ePE juvenile: *n* = 11 (6 male, 5 female); NP adult: *n* = 4 (1 male, 3 female); ePE adult: *n* = 4 (1 male, 3 female). *p < 0.05, Student’s t-test.

Because we noted that a higher degree of hemorrhage was required to produce hypotension in juvenile offspring, we considered the possibility that ePE offspring were resistant to hemorrhage due to increased sympathetic activity. Thus, we also measured serum epinephrine and norepinephrine using ELISA. Consistent with hemorrhage data, we found significantly higher serum concentrations of epinephrine in juvenile ePE offspring (411.2 vs. 337.7 pg/ml, *p* < 0.05, Figure 4A). In contrast, ePE did not have an effect on serum epinephrine in adult offspring (Figure 4B). There was no significant effect of ePE on serum norepinephrine concentration in either group (Figures 4C,D).

### 3.4 Heart Rate and Mean Arterial Pressure in Adult Offspring

**FIGURE 5:**
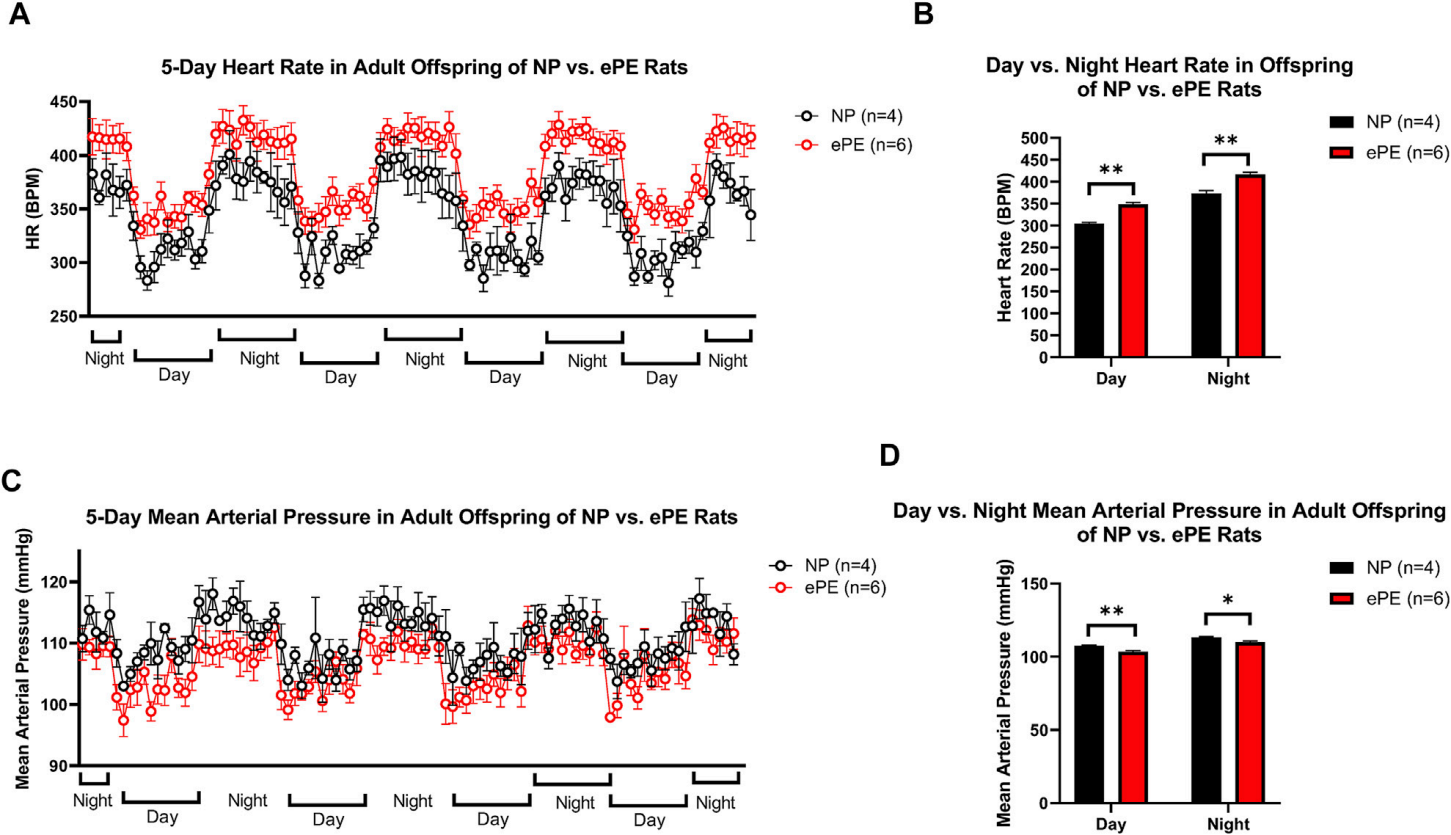
Implanted telemeter data from adult offspring of NP vs. ePE rats. **(A)**, 5 days of continuous telemeter data with heart rates averaged for one-hour periods. **(B)**, a comparison of averages of day (08:00-16:00) and night (20:00-04:00) heart rate for each group. **(C)**, 5 days of continuous telemeter data with mean arterial pressure averaged for one-hour periods. **(D)**, a comparison of averages of day (08:00-16:00) and night (20:00-04:00) mean arterial pressure for each group. Heart rate, but not blood pressure, was persistently elevated in ePE offspring. NP: *n* = 4 (2 male, 2 female); ePE *n* = 6 (3 male, 3 female). **p < 0.01; *p < 0.05, Student’s t-test.

Figure 5 shows continuous HR data and MAP over a 5-day period in adult offspring of NP vs. ePE rats. In panel A, 1-h average heart rates are shown over the course of the experiment, revealing that adult ePE offspring had consistently higher heart rates than their NP counterparts, including higher diurnal and nocturnal HR compared to NP offspring (Figures 5A,B). Despite evidence of elevated heart rate, we did not find a significant elevation in diurnal or nocturnal mean arterial pressure (MAP) in adult ePE offspring, suggesting that the observed elevation in HR was not related to elevated sympathetic tone (Figures 5C,D).

**FIGURE 6:**
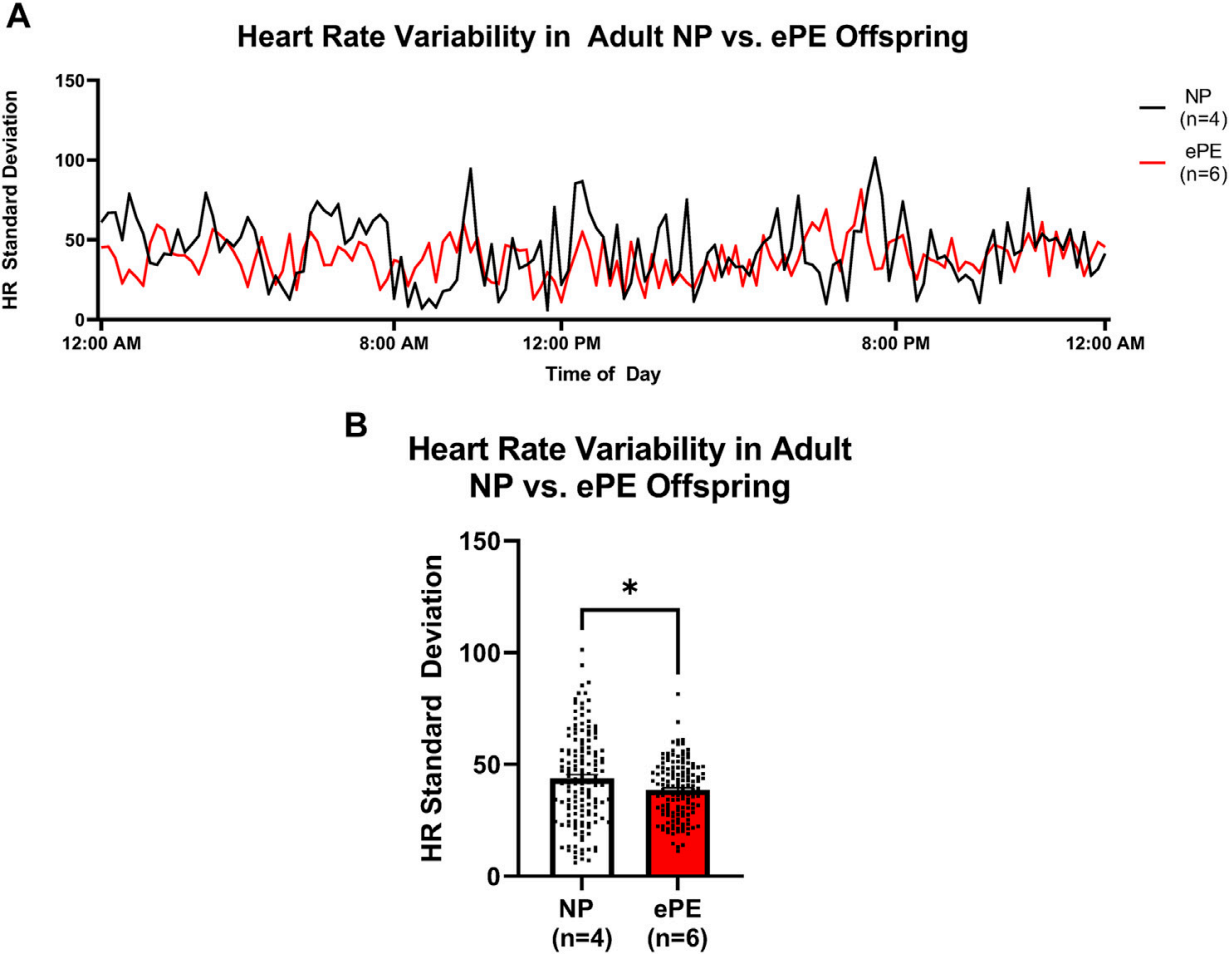
Heart rate variability in adult offspring from NP vs. ePE rats. **(A)**, variability in HR over the final 24 h monitoring period. Data are presented as standard deviation from the mean in 10-min bins for NP (black line, *n* = 4 animals, total of 144 data points over 24 h) vs. ePE (red line, *n* = 6 animals, total of 144 data points over 24 h). Adult NP offspring showed more frequent spikes in variability, and variability during most time periods was higher in NP offspring. **(B)**, average HR variability in adult NP vs. ePE offspring for the entire 24 h period was lower in adult ePE offspring. *p < 0.05, Student’s t-test.

In order to assess potential changes in baroreceptor reflex and/or autonomic control, we assessed the variability of HR over the last 24 h of telemetry recordings (Figure 6). The continuous trace of standard deviation revealed more frequent spikes in variability in NP offspring than ePE offspring during most of the final 24 h of measurement (Figure 6A). When variability was averaged for the entire time period, it was significantly lower in adult ePE offspring (Figure 6B), suggesting baroreflex/autonomic control in offspring from preeclamptic pregnancies may be impaired.

### 3.5 Middle Cerebral Artery Growth and Remodeling

**FIGURE 7:**
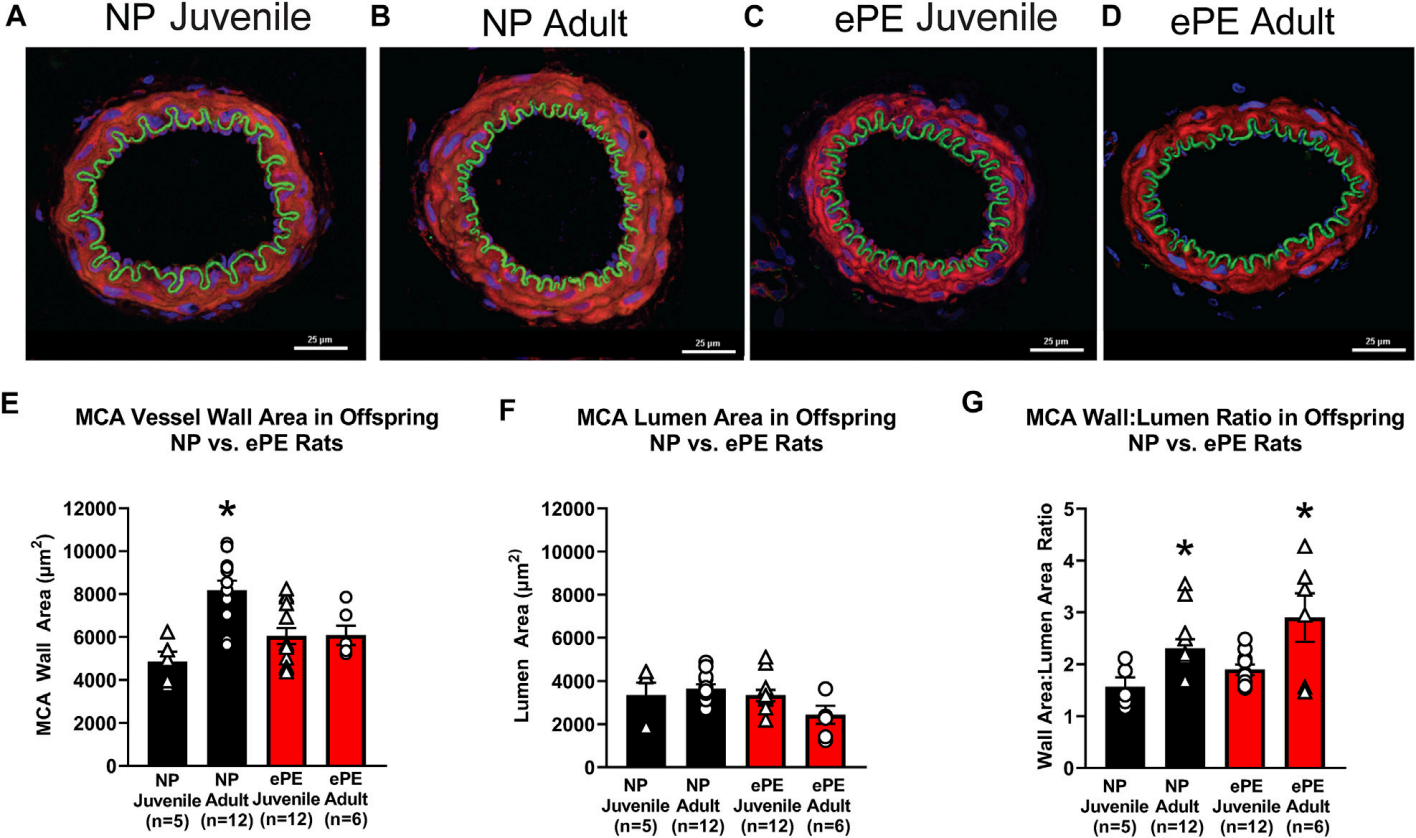
MCA vessel structure in NP vs. ePE juvenile and adult offspring. **(A–D)**, representative images showing size difference in NP, but not ePE offspring over maturation. **(E)**, MCAs from adult NP offspring were significantly larger when compared to juvenile, while those from ePE offspring were not. **(F)**, MCA lumen area did not significantly change over maturation in neither NP or ePE offspring. **(G)**, MCA wall area:lumen area ratio showed an effect of maturation when juvenile and adult offspring were compared. NP juvenile: *n* = 5 (3 male, 2 female); ePE juvenile: *n* = 12 (4 male, 8 female); NP adult: *n* = 12 (6 male, 6 female); ePE adult: *n* = 6 (2 male, 4 female). *p < 0.05; **p < 0.01; 2-way ANOVA with Tukey multiple comparison test.

To assess growth of cerebral arteries over maturation in NP and ePE offspring, immunohistochemistry was used to quantify the area of the medial layer of the MCA as well as lumen area *via* staining for MMP-9. Figures 7A–D show representative images of MCAs from all groups. With respect to MCA medial area, we found a significant effect of aging as well as an interaction of age and ePE (two-way ANOVA, F_1, 34_ = 10.49 and 10.98, respectively, *p* < 0.01; Figure 7E). MCA medial area increased significantly over maturation in NP offspring demonstrating growth with aging from juvenile to adult. However, when comparing offspring from ePE pregnancies, there was no significant increase in medial area, indicating a lack of MCA medial growth. Neither maturation nor ePE had a significant effect on MCA lumen area (Figure 7F). Finally, we found a significant effect of age but not ePE when comparing MCA wall area:lumen area ratio (two-way ANOVA, F_1, 29_ = 13.01, respectively, *p* < 0.05; Figure 7G).

## 4 Discussion

PE leads to increased cardiovascular and cerebrovascular risk in offspring across the entirety of their lifespan (Geelhoed et al., 2010; Fugelseth et al., 2011). The unfavorable intrauterine environment associated with PE exposes the fetus to chronic hypoperfusion, inflammation, and oxidative stress (Bazavilvaso-Rodriguez et al., 2011; Kvehaugen et al., 2011; Aouache et al., 2018; Bharadwaj et al., 2018; Whitaker et al., 2021), all of which may contribute to the development of cardiovascular risk. This increase in cardiovascular risk includes an increased risk of stroke, including perinatal stroke (Wu et al., 2004; Lee et al., 2005; Curry et al., 2007) and stroke in adulthood (Kajantie et al., 2009). It is currently not clear how *in utero* exposure to PE causes increased risk for cardiovascular and cerebrovascular disease in offspring. The present study sought to clarify how exposure to PE affects control of CBF in offspring, and whether changes in CBFAR persist into adulthood.

Regulation of CBF in the brain is distinct when comparing premature, neonatal, infant, adolescent, and adult brain (Rhee et al., 2018). CBF regulation begins *in utero*, and its development continues during the neonatal, infant, and childhood periods (Koehler, 2021). In animal models, multiple studies have found evidence of at least partially functional CBFAR in the fetus (Tweed et al., 1982; Papile et al., 1985; Tweed et al., 1986) and effective CBFAR in the neonate (Leffler et al., 1986; Brady et al., 2007). The study of CBFAR in human neonates is difficult and has largely been limited to those that are anesthetized, critically ill, or both. Nonetheless, available evidence suggest that even premature human neonates exhibit intact CBFAR, and the autoregulatory function becomes more effective as the infant matures (Gilmore et al., 2011; Rhee et al., 2014). Absent disease states or other confounders, CBF meets or exceeds adult levels in children beyond the age of 3 years (Kennedy and Sokoloff, 1957). What remains unclear, however, is when CBFAR reaches functional maturity and how the adverse intrauterine environment affects this important hemodynamic function.

In the present study, we hypothesized that exposure to ePE *in utero* would lead to impairment of CBFAR in juvenile offspring, and that the impairment would persist into adulthood. Contrary to our hypothesis, we found no change in CBFAR curves and no difference in the limits of autoregulation when comparing juvenile offspring from NP and ePE dams. Similarly, there was no effect of ePE on the lower range of autoregulation in adult offspring. However, the upper range of the CBFAR curve was found to be shifted rightward in adult offspring. These findings suggest that both ePE pregnancy and aging have effects on CBFAR in offspring.

We utilized p27–30 rats to assess the effect of ePE on CBFAR in juvenile offspring. While it is not possible to draw direct comparisons between rats and humans, it has been estimated that a p30 rat is similar to an approximately 24-month-old human (Sengupta, 2013; Agoston, 2017). Accordingly, we expected to find functional CBFAR in juvenile offspring from NP pregnancies and impaired CBFAR in juvenile offspring from ePE pregnancies. However, we found nearly identical CBFAR curves in both NP and ePE juvenile offspring, suggesting that ePE does not have a significant effect on CBFAR at the p30 time point. Calculation of upper and lower limits of autoregulation showed an autoregulatory plateau between 38 and 43 mmHg in NP offspring and 39 and 46 mmHg in ePE offspring, which represent a relatively narrow range of pressures over which CBF remains within 20% of baseline. Pryds and colleagues found the lower limit of autoregulation to be 36 mmHg in normocapnic, normoxic 3–5-day-old rat pups anesthetized with isoflurane (Pryds et al., 2005). Our results are consistent with this finding given that the slightly higher lower limit of autoregulation we found is likely due to our use of older, more mature (30-day-old) offspring.

Because brain hypoperfusion (rather than hyperperfusion) in the setting of hypotension is generally of more concern in the very young, relatively little is known about the upper limit of autoregulation in animals of this age. In addition, how the adverse intrauterine environment affects cerebral hemodynamics in offspring, including juvenile and adult, is largely unknown. This study provides the first evidence of which we are aware that exposure to ePE *in utero* causes a shift in autoregulation to higher blood pressures in adulthood. In a prior study, we reported that MCAs from p16 and p23 pups from ePE pregnancies were smaller in diameter and stiffer than those from normal pregnant dams. In addition, MCAs from NP pregnancies enlarged with maturation from p16–p30, a finding that was not present in offspring from ePE pregnancies (Whitaker et al., 2021). Therefore, we hypothesized that CBFAR in ePE offspring would be shifted to the right due to smaller MCAs that could increase resistance to flow and decrease vasodilatory reserve. However, we did not find this to be the case. In adult offspring, CBFAR curves were found to be similar for offspring from both NP and ePE pregnancies over the lower range of blood pressures. However, our study revealed a rightward shift in the CBFAR curve for the upper range of BP with a wider autoregulatory plateau than juvenile offspring: 101–142 mmHg in NP offspring and 106–163 mmHg in ePE offspring. This suggests that in adult offspring from both NP and ePE pregnancies, CBFAR shifts the upper limit to the right by adulthood, regardless of exposure to ePE. It should also be noted that the difference in the upper limit of autoregulation in adult offspring (21 mmHg), though not statistically significant, may have hemodynamic consequences.

In addition to the age-dependent rightward shift to higher pressures, we also found that ePE caused a further rightward shift in adult offspring when compared to NP adult offspring. Though the etiology and physiologic significance of this finding are unclear, it reflects what may be a response to the sequelae of exposure to the unfavorable intrauterine environment of PE that develops over the course of aging. For example, the cerebral vasoconstrictor response to increased blood pressure may be enhanced in adult ePE offspring, thereby shifting the CBFAR curve to higher pressures and protecting the downstream arterioles and microcirculation from vasogenic edema and BBB damage (Iadecola and Davisson, 2008). Multiple previous studies have shown that chronic hypertension is commonly associated with a shift in the CBFAR curve to the right, with both lower and upper limits shifted (Pires et al., 2013). However, in the present study neither NP nor ePE adult offspring demonstrated hypertension. In addition, the rightward shift was only present in the upper limits and not lower, a characteristic distinctly different than chronic hypertension.

In the present study, we found that while MCAs in adult offspring from NP pregnancies had increased medial area, reflecting growth over aging, adult ePE offspring did not. In contrast to our prior study, however, lumen area was not different structurally between groups when measured unpressurized. Although this might suggest that the rightward shift in the upper range of the CBFAR curve in adult ePE offspring was not due to increased resistance to flow, at least as it relates to the unpressurized lumen diameter. However, we analyzed MCA structure in fixed, unpressurized vessels, that may not reflect the *in vivo* state that is highly pressurized. Our group has previously demonstrated an increase in MCA stiffness in ePE offspring at p16 and p23 (Whitaker et al., 2021). Though we did not measure MCA stiffness in the present study, if this stiffness persisted in these older offspring, it may limit the diameters at higher pressures in ePE offspring. The increased stiffness would limit diameter and increase cerebrovascular resistance that could potentially shift the CBFAR to the right. PE is known to increase vascular stiffness in both affected women (Orabona et al., 2017; Phan et al., 2021) and offspring (Plummer et al., 2020). The mechanism of this increase in arterial stiffness remains unknown, but is likely to involve inflammation (Mozos et al., 2017) and/or oxidative stress (Guzik and Touyz, 2017), both of which are present in offspring of ePE pregnancies (Whitaker et al., 2021).

Another possible explanation for the rightward shift in the CBFAR curve in adult ePE offspring is that MCAs in these offspring exhibit increased vascular reactivity (e.g., increased vasoconstriction due to an imbalance of vasodilatory and vasoconstrictive mechanisms). PE is associated with a significant increase in circulating vasoactive substances, including thromboxanes and endothelin-1 that may increase cerebrovascular tone and promote vasoconstriction (Barden et al., 2009; Mousa et al., 2012; Verdonk et al., 2015). In addition, vasculature may be more sensitive to vasoconstrictors in women with PE (Gant et al., 1974). It is unknown whether these features are present in offspring of PE women, but it is likely given that offspring share many of the features that underlie the vasoconstrictive phenotype in their mothers (e.g., inflammation, oxidative stress, endothelial dysfunction) (Ojeda et al., 2011; Whitaker et al., 2021).

While performing controlled hemorrhage during our CBFAR experiments, we noted a relative resistance to hemorrhage-induced systemic hypotension in juvenile, but not adult, offspring from ePE pregnancies. In addition, though ePE did not cause hypertension in adult offspring, we found both diurnal and nocturnal heart rate to be significantly elevated. We hypothesized that this may reflect increased sympathetic activity and an increased ability of the systemic vasculature to contract in response to hypovolemia potentially due to an increased level of and/or increased sensitivity to circulating sympathetic mediators. In fact, we found significantly elevated serum epinephrine in juvenile, but not adult, ePE offspring, and no significant effect of ePE on serum norepinephrine in either age group. The fact that juvenile ePE offspring showed no difference in CBFAR when compared to NP despite elevated serum epinephrine suggests that sympathetic effects have limited influence on CBFAR in juvenile animals. Indeed, prior studies have demonstrated that while the sympathetic nervous system has a role in regulation of CBF in fetuses and premature neonates, its role becomes less prominent as the cerebral vasculature matures and vascular sensitivity to circulating catecholamines and expression of adrenergic receptors diminish over the course of development (Hayashi et al., 1984; Wagerle et al., 1990; Longo et al., 1996). In contrast to juvenile ePE offspring, adult ePE offspring did not show elevated serum epinephrine or norepinephrine. This finding, coupled with the knowledge that the sympathetic nervous system has limited effects on CBFAR in adult animals, suggests that the mechanism of the right shift in the CBFAR curve at higher pressures was not related to sympathetic nervous system dysfunction, at least as investigated in the current study.

Normal hemodynamic responses are the product of a balance in sympathetic and parasympathetic activity. To further probe the autonomic effects of ePE on adult offspring, we assessed HR variability. HR variability (in particular, low HR variability) is considered to be a general indicator of autonomic dysfunction and has been suggested to be predictive of cardiovascular disease risk (Chandra et al., 2012). We found an overall decrease in HR variability in adult ePE offspring, suggesting autonomic dysfunction. Given that these animals also demonstrated elevated HR but not elevated serum epinephrine/norepinephrine, it is possible that decreased parasympathetic activity, rather than increased sympathetic activity, is responsible for our findings. Autonomic dysfunction, including decreased HR variability, is a prominent feature of PE in pregnant women (Yousif et al., 2019; Rueda-Ochoa et al., 2021) and is characterized in PE women by reduced cardiac parasympathetic activity and elevated cardiac sympathetic activity (Eneroth and Storck, 1998; Lewinsky and Riskin-Mashiah, 1998). Though it is currently unknown whether *in utero* exposure to PE leads to autonomic dysfunction in offspring, our study suggests that ePE leads to an imbalance in autonomic nervous system activity that could contribute to increased risk of cardiovascular disease and stroke in adult offspring.

Offspring from PE pregnancies are likely to present for medical care one or more times over the course of their lifespan, potentially in the setting of life-threatening medical emergencies (e.g., acute stroke, hypertensive crisis). It is critical to understand how exposure to PE *in utero* affects cerebrovascular structure and function to ensure optimal cerebral perfusion during medical care. The present study provides the first insights into how PE exposure affects CBFAR in offspring. Here, we demonstrated that exposure to an unfavorable intrauterine environment during ePE had long-term effects on the cerebral vasculature, preventing normal growth and remodeling with aging from the juvenile period to adulthood. This may have contributed to the shift in the upper range of CBFAR to higher pressures in adult ePE offspring compared to adult offspring from NP dams. Further study is required to elucidate the mechanisms of the changes we demonstrated, as well as the downstream effects thereof in order to identify strategies for prevention and/or treatment of CBFAR impairment.

## Data Availability

The raw data supporting the conclusion of this article will be made available by the authors, without undue reservation.
